# Multiple system atrophy

**DOI:** 10.1136/pn-2020-002797

**Published:** 2023-03-16

**Authors:** Yee Yen Goh, Emma Saunders, Samantha Pavey, Emma Rushton, Niall Quinn, Henry Houlden, Viorica Chelban

**Affiliations:** 1 Neuromuscular Diseases, UCL Queen Square Institute of Neurology, London, UK; 2 Multiple System Atrophy Trust, London, UK; 3 Neurobiology and Medical Genetics Laboratory, “Nicolae Testemitanu” State University of Medicine and Pharmacy, Chisinau, Moldova

**Keywords:** MULTISYSTEM ATROPHY, MOVEMENT DISORDERS, CEREBELLAR DEGENERATION, CLINICAL NEUROLOGY

## Abstract

This is a practical guide to diagnosing and managing multiple system atrophy (MSA). We explain the newly published Movement Disorders Society Consensus Diagnostic Criteria, which include new ‘Clinically Established MSA’ and ‘Possible Prodromal MSA’ categories, hopefully reducing time to diagnosis. We then highlight the key clinical features of MSA to aid diagnosis. We include a list of MSA mimics with suggested methods of differentiation from MSA. Lastly, we discuss practical symptom management in people living with MSA, including balancing side effects, with the ultimate aim of improving quality of life.

## Introduction

Multiple system atrophy (MSA) is a sporadic, progressive, adult-onset (>30 years), degenerative disease that presents with a combination of parkinsonism, cerebellar and autonomic dysfunction. Its diagnosis is challenging and postmortem studies show accuracy rates of only 62%–79%.^1 2^ Delayed diagnosis is common, with an average of 3.8 years from symptom onset to diagnosis.^3^ As average survival is only 7–9 years, early diagnosis is crucial for patient quality of life and effective management.^4^

This review aims to discuss the practicalities of establishing a clinical diagnosis, relevant investigations and management of patients.

## What is MSA?

Many terms have previously been used to describe this condition. Graham and Oppenheimer introduced ‘MSA’ in 1969 to encompass multiple neurological ‘entities’ such as olivopontocerebellar atrophy, striatonigral degeneration and Shy-Drager syndrome.^5^

**Figure 1:**
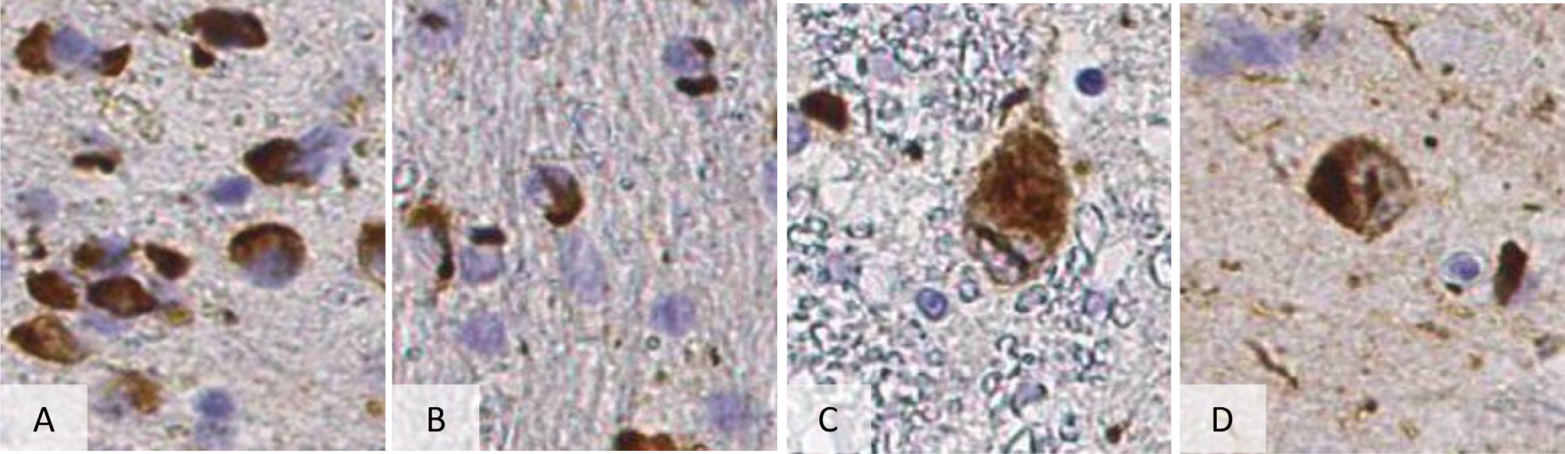
Pathological hallmarks of multiple system atrophy. (A) shows the most frequent α-synuclein positive inclusions are glial cytoplasmic inclusions. There are also glial intranuclear inclusions (as seen in B) to a much lesser extent. However, there are also neuronal cytoplasmic and intranuclear inclusions as seen in C, D. The glial cytoplasmic inclusion density in the grey matter regions, correlates with the extent of neuronal atrophy and gliosis.

Neuropathologically, MSA is a synucleinopathy, characterised by α-synuclein-positive oligodendrocytic glial cytoplasmic inclusions, also known as Papp-Lantos bodies figure 1 .^4^ Striatonigral degeneration and olivopontocerebellar atrophy are self-descriptive neuropathological observations used to classify the main pathological subtypes of MSA.

Clinically, the condition is categorised into MSA-parkinsonian variant (MSA-P) and MSA-cerebellar variant (MSA-C), based on the predominant motor phenotype. MSA-P is associated with striatonigral degeneration pathology and MSA-C with olivopontocerebellar atrophy, although most patients have mixed clinical and pathological features.

Box 1 **Worldwide epidemiology of multiple system atrophy (MSA)^4^**

- Occurs worldwide.
- Incidence: 0.6–3 per 100 000 people per year.
- Prevalence: 1.9–4.9 per 100 000 people.
- Sexes equally affected.
- Onset typically in sixth decade.
- Mean age of onset 56±9 years.
- Relative frequency of motor subtypes differs by geographical and ethnic regions:
  - MSA-cerebellar more in Japanese, Korean and Mestizo populations (70%–80%).
  - MSA-parkinsonian more in European and North American populations (67%–84%).

## Diagnosing MSA

### When to think of it

MSA patients can present to almost any branch of neurology, and often have consulted urology, gynaecology or cardiology before coming to neurology. We recommend considering the diagnosis for any adult with:

- Parkinsonism or ataxia.
- Autonomic dysfunction.
- Atypical resting/action tremor with a myoclonic component.
- Mixed dysphonia with elements of hypophonia, cerebellar dysarthria and spasticity.
- Abnormal posturing with Pisa syndrome or disproportionate antecollis.
- Rapid eye movement (REM) sleep behaviour disorder.

### Diagnostic criteria

**Figure 2:**
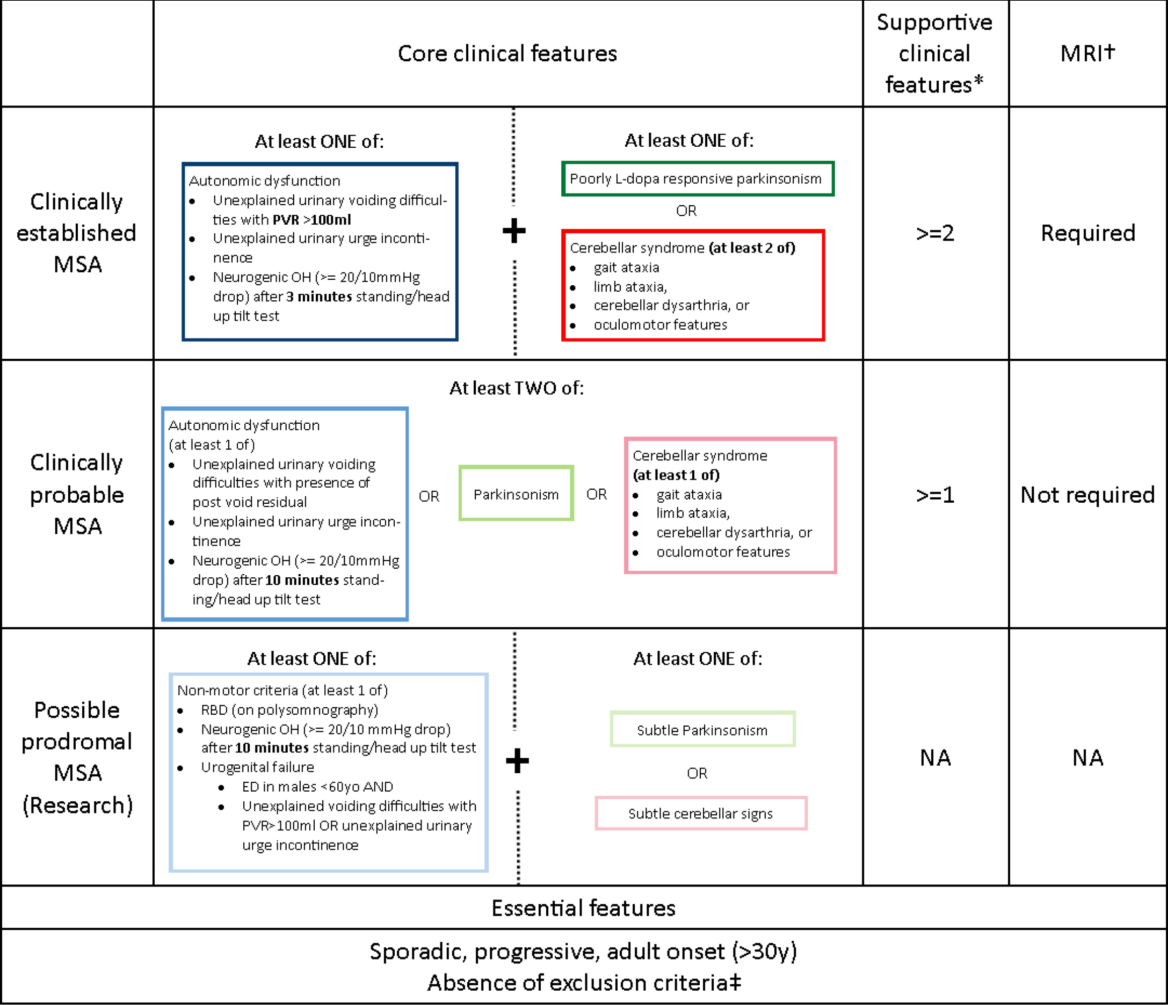
2022 MDS criteria for clinically diagnosed MSA. * †‡See tables 1–3 for details. MSA, multiple system atrophy; PVR,post-voiding residual volume.

In April 2022, the Movement Disorders Society published new diagnostic criteria for MSA defining four levels of certainty: neuropathologically (postmortem) established, clinically established, clinically probable and possible prodromal MSA.^6^ Figure 2 and tables 1–3 provide a visual aid to describe the core clinical criteria, supportive clinical criteria, brain MRI markers and exclusion criteria to make a diagnosis.

**Table 1:** Supportive clinical features^6^

Supportive motor features	Supportive non-motor features
Within 3 years of motor onset evidence of: Rapid progression Moderate to severe postural instability Severe speech impairment Defined as occasional need for repetition during interview Severe dysphagia Defined as dietary modification required Cranio-cervical dystonia induced or exacerbated by L-dopa (without limb dyskinesia) Unexplained Babinski sign Jerky myoclonic postural/kinetic tremor Postural deformities	Stridor Inspiratory sighs Cold discoloured hands and feet Erectile dysfunction (<60 years old) Pathological laughter or crying

**Table 2:** Brain MRI markers of MSA diagnosis^6^

Changes seen	Areas involved for MSA-P	Areas involved for MSA-C
Atrophy	Putamen Middle cerebellar peduncle Pons Cerebellum	Putamen Middle cerebellar peduncle Pons
Increased diffusivity	Putamen Middle cerebellar peduncle	Putamen
Decreased signal on iron-sensitive sequences	Putaminal rim	
T2 hyperintensity	‘Hot-cross bun’ sign in pons	

MSA, multiple system atrophy; MSA-C, MSA-cerebellar; MSA-P, MSA-parkinsonian.

**Table 3:** Exclusion criteria^6^

Exclusion criteria	Clinically established/ probable MSA	Possible prodromal MSA	Differential
Substantial and persistent beneficial response to dopaminergic medications	✓	✗	Parkinson’s disease
Unexplained anosmia on olfactory testing	✓	✓	
Fluctuating cognition with pronounced variation in attention and alertness and early decline in visuo-perceptual abilities	✓	✓	Dementia with Lewy bodies
Recurrent visual hallucinations not induced by drugs within 3 years of disease onset	✓	✓	
DSM-V Dementia within 3 years of disease onset	✓	✓	Dementia with Lewy bodies Progressive supranuclear palsy Corticobasal degeneration
Downgaze supranuclear palsy/slowing of vertical saccades	✓	✓	Progressive supranuclear palsy
Brain MRI findings suggesting an alternative diagnosis (eg, progressive supranuclear palsy, multiple sclerosis, vascular parkinsonism, symptomatic cerebellar disease)	✓	✓	
Documentation of an alternative condition (MSA look-alike, including genetic or symptomatic ataxia and parkinsonism) known to produce similar symptoms and plausibly connected	✓	✓	

DSM-V, Diagnostic and Statistical Manual of Mental Disorders; MSA, multiple system atrophy.

#### The overall criteria are self-explanatory but please note a few points

First, the orthostatic hypotension criteria are now formally specified to be neurogenic. In clinic, this is determined using the Δheart rate (HR)/Δsystolic blood pressure ratio following a 3 min stand (while not taking chronotropic medication). A ratio of <0.5 indicates neurogenic orthostatic hypotension. Where available, continuous cardiovascular monitoring during head up tilt/Valsalva can be used.

Second, a poor L-dopa response is defined either by patient history or by a <30% improvement on the Movement Disorder Society Revision of the Unified Parkinson's Disease Rating Scale part III (MDS-UPDRS III) (not Unified Multiple System Atrophy Rating Scale UMSARS) score while taking up to 1 g of L-dopa (total daily dose) as needed/tolerated for at least 1 month. The acute levodopa challenge has poor accuracy, and a negative result does not exclude the need for a longer trial.

### Key clinical features of MSA

Here, we discuss common history and examination findings in MSA, as well as useful clinical details to improve diagnostic specificity, summarised in table 4A–C.

**Table 4:** (A) Key motor features in MSA. (B) Key autonomic features in MSA. (C) Other important clinical features of MSA

Key clinical feature	Important information
(A)	
Motor features	
Parkinsonism	Often symmetrical, with bradykinesia, rigidity and postural instability. 30%–50% have transient benefit with L-dopa, lasting up to 3.5 years, however, are more susceptible to orthostatic hypotension side effects.^23 35^ L-dopa-induced dyskinesias are often dystonic, affecting the cranio-cervical region, rather than limbs.^36^
Cerebellar syndrome	Manifests as gait ataxia, limb ataxia, cerebellar dysarthria and oculomotor signs (eg, ocular dysmetria, saccadic pursuit, sustained gaze-evoked nystagmus, positional downbeat nystagmus).
Tremor	Patients often have a mixed tremor profile with >1 type of tremor Only 8%–12% have a typical Parkinson’s disease (PD) ‘pill-rolling’ tremor, while around half have a ‘jerky postural tremor’ with polyminimyoclonus.^37^ Up to 35% have a resting tremor, which is often atypical (fast, irregular, myoclonic and remains present on action).^37^ Cerebellar patients can have an intention tremor.
Bulbar dysfunction	Mixed dysarthria with hypokinetic, spastic and ataxic components.^38^ Dysphagia is usually symptomatic within 5 years of motor onset, but silent aspiration probably precedes this.^39^ MSA-P patients often have more severe dysphagia requiring earlier dietary modification, but time to tube feeding is no different.^40^ Sialorrhoea is extremely common, and likely related to oral hypokinesia or pharyngeal dystonia.^39^
Other motor features	Up to 61% of MSA patients show pyramidal signs, for example, upgoing Babinski and hyperreflexia^26^ Abnormal dystonic posturing can occur with Pisa syndrome, disproportionate antecollis and hand/foot contractures. Although uncommon, specificity of these findings is high for MSA vs PD (96.6%–99.2%)^41^ Recurrent falls within 3 years of motor onset are a potential red flag for MSA compared with PD, with a specificity of >97%.^41^
(B)	
Neurogenic orthostatic hypotension	Decreased standing BP by at least 20/10 mm Hg after 3 min, unrelated to cardiogenic causes or hypovolaemia. Manifests as syncope, light headedness or coat-hanger pain (suboccipital and paracervical pain).^42^ Worse in the early morning and hot environments.
Postprandial hypotension	BP drop postmeal (particularly if carbohydrate rich) due to splanchnic vasodilation. Manifests as postural symptoms or generalised fatigue usually within 30–60 min postmeal, but up to 2 hours.^43^
Urogenital involvement	Urinary symptoms can begin years before motor symptoms.^44^ In men, erectile dysfunction often precedes urinary symptoms.^45^ Often, storage symptoms (frequency/urgency±urge incontinence) precede voiding symptoms (reduced stream/flow, dribbling).^46 47^ 8% require catheterisation within 3 years, and 16% over their lifetimes.^24^
Gastrointestinal involvement	Typically, MSA patients present with constipation and delayed gastric emptying, similar to PD, which precedes motor symptoms.
Sudomotor dysfunction	Heat intolerance or flushing due to progressive hypohidrosis, which is more severe in MSA than in Parkinson’s disease.^48^ Impaired peripheral vasomotor function/ circulation leading to ‘reddish-blue’ discolouration of the extremities, often associated with the ‘cold hand’ or ‘cold foot’ sign.^49^
(C)	
Respiratory symptoms	Stridor (13%–69% of all MSA patients)^50^ Defined as a strained, high-pitched, harsh inspiratory sound, due to vocal cord paralysis or dystonia in MSA). Typically begins at night. Patients are often not aware themselves, but caregivers will often report a ‘peculiar snore’. It can be useful to ask for a video/audio recording of this, but otherwise video polysomnography can differentiate it from oropharyngeal snoring Laryngospasm.^51^ Sudden, prolonged, forceful apposition of the vocal cords causing sudden suffocation or stridor and is a medical emergency if persistent. Central respiratory insufficiency.^52^ MSA patients may have impaired ventilatory drive with minimal to no chemosensitivity to hypoxia, abnormal respiratory rhythm during sleep and central hypoventilation, some of which may explain sudden death in MSA (despite tracheostomy).
Sleep-related symptoms	REM sleep behaviour disorder – in up to 81% of MSA patients.^52^ The most common sleep-related disorder in MSA; can precede motor symptoms by years.^53^ It is characterised by sleep-related vocalisations and often violent complex motor behaviour during REM sleep. The gold standard diagnosis is polysomnography showing loss of atonia during REM sleep; Where polysomnography is unavailable, standardised questionnaires for example, Innsbruck REM sleep behaviour disorder inventory may help^54^ Periodic limb movements—in 44%–60% of MSA patients.^55^ Defined as repetitive movements of the limbs during sleep, of which patients are typically unaware, but sleep quality is affected. Restless legs syndrome—in up to 28% of MSA patients^56^ This is a sensorimotor disorder characterised by an urge to move the limbs, often with paraesthesia or pain. Symptoms are more noticeable in the evenings or when inactive. Associated with low serum iron concentrations.
Cognition	Up to 49% of MSA patients have some executive dysfunction on in-depth testing.^57^ Like progressive supranuclear palsy, MSA patients can report emotional lability with pathological laughter or crying, although their symptoms are less severe, and without memory difficulties.^58–60^ Unlike PD, well-formed visual hallucinations and fluctuating cognition are infrequent in MSA.^61^
Miscellaneous	Anosmia is not typical of MSA, and might instead indicate PD.^45^ Intermittent diplopia can occur due to midbrain dysfunction.^62^

BP, blood pressure; MSA, multiple system atrophy; MSA-P, MSA-parkinsonian.

### History

In clinic, attention is naturally drawn to visible or audible neurological (often motor) deficits (eg, dysarthria or dystonic posturing) but it is important to look for the many non-motor or nocturnal MSA symptoms (see table 4). Things to ask about include mobility and use of aids, urinary (storage/voiding) symptoms, orthostatic symptoms, bulbar symptoms, changes in smell and mood/affect. Patients rarely offer symptoms of erectile dysfunction except on direct questioning.

A timeline of symptom evolution can help to gauge the rate of progression. Recurrent falls, severe speech and severe swallowing changes within 3 years of motor onset would support MSA.^6^

We encourage patients to attend with a caregiver who can provide a collateral history, in particular around sleep (eg,REM sleep behaviour disorder or periodic limb movements of sleep), stridor, obstructive sleep apnoea and cognitive change. With intermittent symptoms such as stridor, audio recordings can be helpful.

### Examination

As in all of neurology, the examination of an MSA patient begins from the waiting room. Patients may have a wide-based or shuffling gait, often with a stooped posture. Retrocollis or a rigid extended posture might suggest progressive supranuclear palsy.

Abnormal postures such as Pisa syndrome (laterocollis of the trunk), disproportionate antecollis or contractures of the hands and feet may indicate MSA. Asymmetrical limb dystonia (or an alien limb) could relate to corticobasal syndrome.

Eye movements should be examined (saccades and pursuit) for cerebellar signs and for vertical saccade slowing/gaze restriction, indicating a supranuclear gaze palsy, as in progressive supranuclear palsy (table 5). A head impulse test is a useful screen particularly in MSA-C patients, looking for evidence of peripheral vestibulopathy as seen in CANVAS (cerebellar ataxia, neuropathy, vestibular areflexia syndrome).

**Table 5:** Provides a list of possible MSA mimics and when to consider them

MSA mimics	Consider if…
Parkinson’s disease	Significant and sustained response to L-dopa (>30% improvement in UPDRS-III), anosmia, delayed onset of autonomic symptoms
Dementia with Lewy bodies^60^	Fluctuating consciousness and cognitive change with hallucination (particularly visual), not secondary to L-dopa treatment
Progressive supranuclear palsy^63^	Vertical saccade slowing, supranuclear gaze palsy Frontalis overactivity and retropulsion Frontal cognitive change, non-fluent variant primary progressive aphasia
Corticobasal degeneration^64^	Asymmetrical limb dystonia, alien limb
Vascular Parkinsonism^65^	Onset >75 years, vascular risk factors, dementia
Normal pressure hydrocephalus	Cognitive impairment, lack of upper limb signs
Neurogenetic conditions	
Spinocerebellar ataxia types 1,2,3,6,7,12,17^66^	May have a positive family history, but deterioration likely slow
CANVAS^67^	Chronic dry cough, positive head impulse test, peripheral neuropathy
C9ORF72 expansion^68^	Positive family history, lower motor neurone signs (eg, fasciculation, wasting)
Friedreich’s ataxia^66^	Peripheral neuropathy, pes cavus, loss of lower limb reflexes cardiomyopathy, type two diabetes mellitus
Fragile X tremor–ataxia syndrome^66^	X-linked inheritance, peripheral neuropathy, behavioural disorders with executive dysfunction

CANVAS, cerebellar ataxia, neuropathy, vestibular areflexia syndrome; MSA, multiple system atrophy; UPDRS-III, Unified Parkinson’s Disease Rating Scale part III.

MSA patients often (but not always) have symmetrical bradykinesia and rigidity. Tremor should be examined at rest, with an outstretched posture and with action. Look for myoclonus (spontaneous/stimulus induced) or polyminimyoclonus. Cerebellar signs should be elicited through tests for dysdiadochokinesia, finger–nose and heel–shin testing. Tandem gait is often affected early in gait ataxia, but may not be specific for cerebellar dysfunction.

Reflexes and plantar responses should be checked for pyramidal involvement. Depressed reflexes with an abnormal sensory examination could indicate a peripheral neuropathy and consideration of CANVAS, Friedreich’s ataxia and fragile X tremor–ataxia syndrome).

All patients should have a lying and standing blood pressure and HR measured if they can stand for 3 min. In addition, retropulsion should be checked unless the patient is highly likely to come to harm from it.

Over the last few years, there have been several case reports of autoimmune antibody mediated disease (CV2/CRMP5, Anti-Hu, Homer-3) causing symptoms in keeping with MSA.^7–9^ However, these cases usually stand out from typical MSA in having with extremely rapid symptom progression, within weeks to months.

### Investigations

#### Neuroimaging

##### MRI

All patients suspected to have MSA should have an MR scan of brain with standard and iron specific sequences. Table 2 summarises typical MRI markers for disease. The well-known pontine ‘hot cross bun’ sign has high specificity (97%), but only 50% sensitivity. The ‘putaminal rim’ sign is a hyperintense rim to the putamen on iron sensitive imaging seen in MSA-P with 90% specificity and 72% sensitivity.^10^

MRI also helps to exclude differentials such as normal-pressure hydrocephalus, vascular disease or white matter changes.

In patients with a normal MR scan of brain but a high clinical suspicion of MSA, we recommend obtaining a DATScan and repeating the MRI 12–18 months later.

##### DatScan

The DATScan is usually abnormal in patients with clinical parkinsonism, and does not differentiate between MSA, Parkinson’s disease or other atypical parkinsonian syndromes.^11^ However, it may be useful when considering conditions like vascular parkinsonism, essential tremor or early MSA-C, where parkinsonian symptoms are subtle.

##### Other supportive imaging

Where the diagnosis is unclear, a fluorodeoxyglucose (FDG)-positron emission tomography (PET) may help by showing hypometabolism in the putamen, brainstem or the cerebellum.^12^ Otherwise, cardiac MIBG scintigraphy can look for postganglionic autonomic dysfunction (not typically found in MSA, ie, a normal scan expected). However, common conditions like diabetes mellitus can have abnormal findings, which clouds the picture. Also, up to 30% of MSA patients can have cardiac MIBG abnormalities, calling into question the validity of this test.^13 14^

##### Autonomic function testing

The only tests needed to fulfil core clinical criteria are a postvoid bladder residual volume and a lying and standing blood pressure. However, if uncertainty remains, other investigations can help to prove autonomic involvement, for example formal urodynamics showing detrusor sphincter dyssynergia^15^ or external anal sphincter electromyography showing chronic reinnervation changes in Onuf’s nucleus (however, this finding is not specific to MSA, and occurs in long-standing Parkinson’s disease, progressive supranuclear palsy and after pelvic surgery).^16^

### Discussing the diagnosis with patients

It can be challenging to share a diagnosis of MSA with a patient and their family. This section covers frequently asked questions, such as ‘How long do I have?’ and ‘What will happen to me over time?’

#### Survival

The average survival from onset for an MSA patient is around 7–9 years.^4^ However, a pathologically confirmed case series of a ‘benign’ MSA variant found survival of >15 years^17^ and the longest reported survival of a neuropathologically confirmed MSA-P case is 23 years from onset.^18^ Conversely, there is a report of an aggressive MSA phenotype with a very short disease duration of <3 years.^19^ Respiratory and urinary tract infections are common causes of death, together with sudden death (probably from central apnoea, epiglottal airway obstruction and/or cardiac dysautonomia).^20^

Overall, there is no simple clinical indicator of prognosis. A meta-analysis of 39 studies (4282 MSA patients) showed a non-significant increased multivariate HR of 1.22 (95% CI 0.83 to 1.80) in patients with severe dysautonomia and early combined autonomic and motor symptoms. Early falls were strongly associated with shorter survival (HR 2.32, 95% CI 1.94 to 2.77) but not with sex. There is conflicting evidence regarding age at onset and stridor.^21^

#### Disease progression

After the onset of motor symptoms, ~30% of patients require walking aids within 3 years, and up to 60% are wheelchair-bound by 5 years and bedbound by 8 years.^22^ At 6 years, ~10% of patients require gastrostomy and have unintelligible speech.^23^ MSA-P patients generally have more functional impairment than those with MSA-C, but the overall rate of progression and survival is no different.^24 25^ In a study published after the previously discussed meta-analysis, early autonomic symptoms were associated with increased rate of disease progression but not of death.^3^

### Management

No disease-modifying treatment has proven so far to alter MSA progression, and thus symptom management remains the mainstay of care. As with any neurodegenerative disease, important principles to consider include:

- Patient and carer education on the practicalities of medication use.
- Regular rationalisation of medication to reduce adverse effects and to improve quality of life.
- Emphasising the importance of non-pharmacological interventions.
- Early referral to allied healthcare professionals to maintain function for as long as possible, emphasising a multidisciplinary team (MDT) approach for care.
- Early discussion around disease progression and advance care planning, as well as timely consideration for referral to palliative care (taking into consideration patient-specific wishes).

Local charities (eg, the MSA Trust (UK) and the MSA Coalition (USA)) are useful sources of information for patients about the disease as well as local services available to patients for example, specialist nursing, social welfare or financial support specific to their needs.

### Symptom management in MSA

#### Motor symptoms

Tables 6–10 summarise our general approach to managing symptoms in MSA.

**Table 6:** General approach to managing motor symptoms in MSA

Symptom	Treatment approach
Parkinsonism	Early referral to physiotherapy and occupational therapy to help maintain independence for as long as possible. All parkinsonian patients should have a slow uptitration L-dopa trial to at least 1 g a day for 3 months. Down-titrate L-dopa medication when/if it is no longer perceived to be effective, but reinstate the last dose if there is a deterioration. In patients with orthostatic hypotension, consider simultaneous midodrine if there are at least 4 hourly intervals between L-dopa doses. Modified release L-dopa can help nocturnal symptoms but be cautious about nocturnal hypotension, in particular in those with nocturia and mobility issues.
Ataxia and vertigo	There is no proven effective pharmacotherapy for this. Early referrals to physiotherapy and occupational therapy. Review medications and stop unnecessary muscle relaxants or daytime sedatives, to help balance. Look for and treat concomitant reversible causes of vertigo.
Falls	Early referrals to physiotherapy and occupational therapy . Consider DEXA scanning and FRAX score calculation and manage for osteoporosis
Dystonia	Determine whether dystonia is secondary to levodopa-induced dyskinesia (ie, increases at peak dose); if so adjust medication accordingly Treatment naïve/wearing off dystonia may respond to levodopa. Focal dystonia can respond to botulinum toxin injections, while generalised dystonia may respond to clonazepam 0.5–1 mg or baclofen 5 mg three times a day. This should be done in conjunction with physiotherapy for splinting Anticholinergics can be tried, but autonomic adverse effects may limit their use. Physiotherapy referral for stretching and splinting alongside botulinum toxin can help.

DEXA scan, dual-energy X-ray absorptiometry scan; FRAX, Fracture Risk Assessment Tool; MSA, multiple system atrophy.

**Table 7:** Cardiovascular autonomic dysfunction

Symptom	Treatment approach
Orthostatic hypotension	Non-pharmacological methods should be tried in all patients. Pharmacological treatment includes midodrine (first line) followed by fludrocortisone then pyridostigmine. Regularly review need for pharmacological treatment as progressive loss of mobility decreases the duration of being upright/standing.
Supine hypertension	Elevate head of the bed by at least 30° overnight. Avoid supine positions during the day where possible. The last midodrine dose should be no less than 4 hours before bed. A small carbohydrate-rich meal in the late evening may help. Take a short-acting antihypertensive medication (eg, nifedipine/losartan) before bed.
Postprandial hypotension	Encourage small, low carbohydrate meals but increase the frequency of eating. Time the midodrine dosing around meals. Consider using acarbose and octreotide, although octreotide is only available for prescription in specialist centres for this indication.

**Table 8:** Genitourinary autonomic dysfunction

Symptom	Treatment approach
Urinary storage symptoms	Storage symptoms Modify fluid intake, avoid caffeine, acidic juices and carbonated drinks. The various antimuscarinics have roughly equal efficacy; choose based on local formulary. If needing a less-sedative option, consider Trospium MR 60 mg once daily. Beta 3 agonists (eg, mirabegron) have the added benefit of improving blood pressure (useful for MSA patients) but can exacerbate supine hypertension. Intravesical botulinum toxin or percutaneous tibial nerve stimulation could be considered for resistant cases. Monitor postvoiding residual volume before starting medication. If this approaches 100 mL, repeat 2 weeks after starting medications to look for retention. This may require either stopping the drug or using a catheter Voiding symptoms In men aged over 60 years, consider and examine for prostate enlargement as it commonly contributes to symptoms. If postvoiding residual volume is >100, consider intermittent self-catheterisation by patient/carer at least 3–4 times daily in the first instance. Consider a long-term catheter in patients who cannot manage intermittent self-catheterisation. Consider a suprapubic catheter early.
Nocturia	Reduce fluid intake in the 3–4 hours before bedtime. An evening dosing of anticholinergic can help some patients. Intranasal desmopressin can be considered If needed but requires regular monitoring of serum sodium due to its hyponatraemic adverse effects. If there is a concurrent risk of nocturnal hypotension, consider a convene, bottles or pads to reduce night-time mobility.
Erectile dysfunction	Consider that this could be due both to autonomic dysfunction but also the psychological impact of disease, and treat accordingly. Phosphodiesterase 5 inhibitors work well, but often cause hypotension, limiting their effectiveness.

MSA, multiple system atrophy.

**Table 9:** Bulbar symptoms

Symptom	Treatment approach
Sialorrhoea	Off label use of atropine 1% eye-drops (but taken orally) two drops as needed but up to three times daily should be first line treatment in patients able to self-administer or communicate their needs. Alternatively, an ipratropium bromide inhaler (1–2 puffs up to four times a day) can be used off label. Hyoscine hydrobromide patches and subcutaneous glycopyrronium bromide can help, but often patients need 12-weekly botulinum toxin injections for symptom relief.
Speech	Early referral to speech and language therapy is important to preserve communication (eg, with voice therapy or speech adjuncts) for as long as possible and to avoid missing opportunities such as voice banking. The MSA Trust provides funding for voice banking for their members living in the UK and Ireland and patients should be directed to this early.
Swallowing	Consider early speech and language therapy referral to promote patient safety and quality of life. There is no evidence that gastrostomy reduces risk of aspiration/increases survival in patients, but it can be considered a valid measure to improve quality of life (reducing meal anxiety, medication burden and improving hydration status).

MSA, multiple system atrophy.

**Table 10:** Respiratory and sleep

Symptom	Treatment approach
Stridor and other respiratory issues	Referral to ENT for laryngoscopy in the first instance to exclude mechanical lesions or other secondary causes of vocal cord dysfunction. Urgent referral for patients reporting laryngospasm. Drug-induced sleep endoscopy or video polysomnography if awake investigations show no cause.^50^ CPAP therapy reduces stridor symptoms, but with no evidence of increased survival.^50^ Tracheostomy eliminates stridor symptoms, and may increase survival, but evidence for this is weak.^50^
Sleep-related syndromes	REM sleep behaviour disorder. Discuss creating a safe-sleep environment for patient and partner. If medication needed for MSA patients, first line is melatonin and second line is clonazepam, owing to potential drowsiness and stridor.^34^ Obstructive sleep apnoea. If symptoms interfere with quality of life or Epworth scores >9, refer to a sleep specialist. Restless legs syndrome. Check serum iron concentrations in all patients and replace iron as appropriate. For ongoing symptoms treat with gabapentin/pregabalin or consider a dopamine agonist (eg, ropinirole).

CPAP, continuous positive airway pressure; ENT, ear, nose and throat; MSA, multiple system atrophy; REM, rapid eye movement .

Although the 2022 MSA diagnostic criteria suggest a chronic levodopa trial (starting 62.5 mg three times a day, increasing by 62.5–125 mg every 2 weeks, until reaching 250 mg four times a day) for 1 month, in practice, we often give it for 3–6 months before reviewing for benefit. Crucially, patients should be informed as to which symptoms the medication is aiming to treat, to best review its effects.

Patients who develop nausea with levodopa can be prescribed domperidone 10 mg three times a day preferably for short term (1–2 weeks) use during initiation. Before the prescription, patients should have a baseline 12-lead ECG, noting that a corrected QT interval (QTc) of >450 ms (in men) and >470 ms (in women) is a contraindication to its prescription. If domperidone needs to be continued long term, it is important to discuss possible cardiac adverse effects with the patient. Trials of dose reduction should be regularly considered. As per previous guidance from the Association of British Neurologists, patients should have a repeat ECG after 2 weeks. Patients should avoid taking a second QTc prolonging medication but if this is unavoidable, consider stopping domperidone or arrange close ECG monitoring.

During dopamine withdrawal, some patients notice deterioration and should be advised to continue the last dose of benefit.

There is weak evidence to suggest that levodopa is more effective than dopamine agonists in MSA. Given the additional higher risk of impulse control disorders with dopamine agonists, we favour using levodopa in the first instance.^26^Focal limb dystonia may respond well to botulinum toxin, but it is important to be cautious when treating cervical dystonia or antecollis, as injection around the deep neck flexors can cause quite significant dysphagia. With antecollis and laterocollis, patients often find support cushions (eg, flight neck support) or pillows helpful.

#### Management of cardiovascular autonomic dysfunction

In the first instance, we encourage patients to own a home blood pressure machine to individualise their management better. Non-pharmacological management should be prioritised.

Best evidence for pharmacological treatment of neurogenic orthostatic hypotension is for midodrine,^27^ an alpha agonist that increases the blood pressure through vasoconstriction. It is started at 2.5 mg three times a day, with gradual up-titration by 2.5 mg weekly to 10 mg three times a day as required symptomatically. Adverse effects include dose-dependent supine hypertension and scalp itching. It is contraindicated in patients with cardiac conduction defects and high vascular risk, for example, myocardial infarctions and ischaemic stroke.^27^ Patients taking midodrine should be counselled to stay upright after its use to avoid supine hypertension; if they foresee a need to lie down, they can miss the preceding dose. The last dose of midodrine should be at least 4 hours before bed to reduce risk of supine hypertension.

There is only limited evidence around fludrocortisone and pyridostigmine, but if midodrine is unsuitable they are worth trying. Fludrocortisone is a mineralocorticoid that increases blood pressure through volume expansion. It is started at 0.1 mg in the mornings and increased weekly by 0.1 mg as required for symptoms to a maximum of 0.4 mg. It carries a risk of nocturnal hypertension and monitoring blood pressure at night is useful in the first 2–3 weeks after a dose change. Other adverse effects include fluid retention and hypokalaemia.

Pyridostigmine probably works by increasing acetylcholine availability at autonomic ganglia involved in the baroreflex, thus increasing the blood pressure without supine hypertension. It can be started to 30 mg three times a day and increased weekly by 30 mg to a maximum daily dose of 360 mg. It is not as effective as fludrocortisone but is worth a trial in patients with severe symptoms.^27 28^

Low-quality evidence suggests that fluoxetine, domperidone, atomoxetine and yohimbine may help with orthostatic hypotension.^27^ These should not be considered as direct orthostatic hypotension management, but if required for their primary use (eg, as an antidepressant/antiemetic), their selection may help some patients.

Droxidopa is available for prescription outside the UK and European Union. This is a prodrug for norepinephrine, which increases standing systolic blood pressure through vasoconstriction. Short placebo-controlled clinical trials have shown improvement in dizziness and in standing blood pressure, with post-hoc analysis suggesting fewer falls. However, importantly, two phase 3 trials did not reach their primary outcome of improvement in the Orthostatic Hypotension Questionnaire score, and further evidence suggests there is reduced clinical effectiveness after the first few weeks of treatment.^29^ The RESTORE trial (completed July 2022) will shed further light on the long-term (12-month) effectiveness of droxidopa.

With supine hypertension, we aim for a systolic blood pressure of <180 mm Hg when lying flat. If medication is needed, we start a low dose at night (5 mg nifedipine/25 mg losartan) but are cautious of hypotension, particularly if patients need to mobilise at night for nocturia. Other pharmacological options include clonidine (0.1–0.2 mg) or glyceryl trinitrate patches (0.1–0.2 mg/hour).^30^

#### Genitourinary symptoms

Before starting treatment for urinary storage symptoms, we recommend checking a postvoid volume, and if approaching 100 mL, repeating this 2 weeks after starting medication. Patients who do not respond to oral medication with no evidence of active infection, can be considered for intravesical botulinum injection, but patients should be warned that they may need catheterisation after treatment.

Many MSA patients with voiding problems (eg, postvoid volume of >100 mL) initially use intermittent catheterisation. However, with time, reduced hand dexterity and insertion difficulty can cause pain and urinary tract infections. The advantages ofsuprapubic catheterinclude improved comfort and no risk of urethral trauma/erosion. They are also less likely to get blocked as larger bore tubes can be inserted. Thus, we generally recommend suprapubic catheters to our patients. Although low level evidence suggests that bacteriuria is reduced, there is no evidence that they cause fewer infections and there is a possibility of urethral leakage.^31^

Nocturia is common in people with MSA. In patients who practise intermittent self-catheterisation we recommend emptying of bladder completely just before bed. Anti-muscarinics can help (when unrelated to nocturnal polyuria) but may cause sedation that could lead to overnight falls. There is no evidence to support any anti-muscarinic over another, and we recommend treating according to local formulary guidance.

MSA patients can have nocturnal polyuria (>33% of daily urine production occurring overnight) due to reduced daytime renal perfusion pressures with orthostatic hypotension. With this, intranasal desmopressin (10 mg at night) can help, but patients need monitoring for hyponatraemia at baseline, 1 week post -dose adjustment, 1 month and 3 monthly thereafter if stable.

#### Bulbar symptoms

With sialorrhoea, atropine and ipratropium bromide usually work well as patients can adjust their dosing and timing. If it is insufficient, we normally recommend half a hyoscine patch for 72 hours for a couple of weeks, before increasing to a full patch if required. In patients without dysphagia or orofacial incoordination/dystonia, gum chewing can increase swallow frequency and so help with hypersalivation.^32^

We believe it is important to make early referral to speech and language therapy services for voice therapy (eg, Lee Silverman) and to consider options such as voice banking. Voice banking is the process of creating a personalised synthetic voice, to use later. Patients and carers often are not aware of the availability of communication aids (eg, amplifiers, tablets or eye-gaze devices) and may not think to approach healthcare professionals for assistance with communication, making it important for the MDT to offer these services proactively.

Swallowing difficulties often cause significant distress in people with MSA. Frequent choking can cause meal anxiety and increased meal duration (sometimes up to 1–2 hours) reduces quality of life. Dysphagia of liquids also has implications for fluid status, and for ability to participate meaningfully with everyday activities. Again, we recommend early referral to speech and language therapy. This is partly for support around textures and positioning, but also because the change in swallowing and diet often marks a noticeable change in a patient’s life, and can trigger thoughts around disease progression and planning. Not everyone will want to start those conversations then; however, it is a good time to make people aware of their options (eg, risk feeding, gastrostomy) and to know that there is a team which whom they can to discuss these should they wish.

There is insufficient evidence on the best timing for gastrostomy insertion. On balance, we recommend that this be done before the patient is in extremis; in our experience urgent admissions, are often preceded by malnutrition and dehydration giving greater procedure risk, as well as increased patient distress. Once the tube is in, the patient can to eat and drink for pleasure if they wish but use the tube for nutrition and fluids. There is no evidence that gastrotomies reduce aspiration or infections, but they are a potential palliative measure to improve the quality of the life.

#### Respiratory and sleep symptoms

Patients and their carers are often understandably worried about stridor. Its treatment with non-invasive ventilation±tracheostomy is usually decided between the patient, their family and the sleep/ENT team. However, a pretreatment video-fluoroscopy of the epiglottis is useful, as MSA patients (up to 71%) can have a floppy epiglottis, which can be moved by the air pressure thus blocking the trachea.^33^ There is only weak evidence for long-term benefit of either non-invasive ventilation or tracheotomy, and it is important that patients/families understand this; the main aim of treatment is for symptomatic benefit, and the presence of a tracheostomy or non-invasive ventilation machine does not prevent sudden death.

All patients with REM sleep behaviour disorder need a safe sleep environment. Depending on severity, consider lowering mattresses, placing padded mats on the floor and shifting bedside tables. Bedpartners may need separate beds to avoid injury.

There is only limited good quality evidence for pharmacological treatment of REM-sleep behaviour disorder. Melatonin may be less effective than clonazepam in idiopathic REM-sleep behaviour disorder, but given the other respiratory concerns in MSA (eg, obstructive sleep apnoea, hypoventilation) we advise melatonin in the first instance.^34^

#### Mood and cognition

##### Mood and affect

When treating mood or anxiety disorders in MSA no specific medication class has greater or lesser efficacy, and treatment should be in keeping with local/national guidelines. However, consider medication secondary effects when choosing medications. For example, limited evidence suggests that fluoxetine helps with orthostatic hypotension. Similarly, anticholinergic adverse effects of tricyclic antidepressants may limit their use in MSA patients.

Historically, pathological crying and laughing has been treated with the use of selective serotonin reuptake inhibitors (SSRIs) and serotonin and norepinephrine reuptake inhibitors (SNRIs), although these medications have unknown effectiveness. Dextromethorphan/quinidine (Nuedexta) is not commercially available in Europe for this but is available in the USA.^33^

Simple acknowledgement of symptoms as part of the disease can often help to alleviate patient and carer concerns around this, as can other interventions such as respite care or carer psychological support.

##### Hallucination and psychosis

Severe cognitive involvement is rare in MSA, although it does not exclude the diagnosis. If prominent, consider alternative diagnoses for example, dementia with Lewy bodies, Parkinson’s disease dementia or drug-induced psychosis, each of which has its own management. If required, quetiapine or olanzapine can help, especially in urgent situations, but this must be weighed against possible drug-induced parkinsonism.

Clozapine is effective in patients with Parkinson’s but needs regular monitoring for agranulocytosis, often best done through already established mental health services.

##### Advanced MSA

MSA progression is inevitable, and issues arising in the last 1–2 years of life can often be anticipated. Early palliative care involvement often helps patients and their families manage their disease and advance care planning. Table 11 summarises several issues discussed in the last section regarding advance care planning in MSA.

**Table 11:** Planning in advanced MSA

Symptom	Treatment options	When recommended	Things to consider	Comments
Swallowing difficulties; Inability to maintain sufficient nutrition/hydration, repeated aspiration pneumonia or choking	Diet modification and supplementation, risk feeding, gastrostomy insertion	When unable to maintain sufficient oral intake to maintain weight, when swallow is unsafe, if chest infections/aspiration pneumonia. Allow time for patient to consider pros and cons and assist patient to document wishes.	Can improve quality of life and help manage blood pressure, hydration, constipation, and may not be possible in later stages	Percutaneous endoscopic gastrostomy (PEG) may improve quality but not length of life. There is currently insufficient evidence on PEG or radiologically inserted gastrostomy use in MSA.
Speech and communication difficulties	Low-tech equipment options can help communication access via speech and language therapy (SaLT)—a specific communication assessment may need to be requested.	Voice banking as early as possible (can be done through MSA Trust and SaLT). Regular review by SaLT to assess changes to communication and allow time to refer onto specialist services if required.	Equipment can be useful and aid communication but use of technology needs to be learnt in good time. It can greatly improve quality of life and reduce isolation. Mobility/dexterity issues and cognitive issues can limit use of communication devices.	NHS England fund Augmentative and Alternative Communication services help people communicate as effectively as possible when speech is impaired. Environmental and access to technology services are also available. Get to know what service is in your area and the referral criteria.
Mobility	Appropriate equipment and support to maintain safety, provided by occupation therapy and physiotherapy and other services as appropriate, for example, tissue viability, wheelchair services, orthotics.	When current methods of mobilising or transferring might become unsafe or uncomfortable. In advanced stages when too fatigued or sleepy to want to be sat out of bed, or when they find they prefer to remain in bed for longer periods.	Patients will need appropriate equipment, support and care to maintain skin integrity and avoid pressure areas developing and meet care needs.	Patients will need support of occupational therapy teams for equipment. Care services may be involved. District Nurses and Community Matron can provide care, facilitate services and be link between patient and GP. Neurology and Specialist services may need to link with GP and specialist palliative care services.
Respiratory symptoms—aspiration, stridor, vocal cord paralysis, obstructive and central sleep apnoea, respiratory insufficiency	Respiratory support such as CPAP may be needed, or further input from respiratory if already in place. Tracheostomy may be considered if CPAP insufficient.	Discuss potential for respiratory difficulties in advance. Refer for investigation and specialist support if symptomatic.	CPAP may not be tolerated or discontinued if infection or daytime respiratory insufficiency. Tracheostomy may have impact on care needs.	Sudden death can still occur even with CPAP or tracheostomy due to central sleep apnoea. Advance care planning should be in place to understand patient’s preferences in good time and to prevent decisions about for example, tracheostomy being made in a crisis or medical emergency.
Recurrent infections	Antibiotics for acute infections, discuss treatment preferences with patient.	As early as possible; consider ‘rescue’ antibiotics at home and remember people with MSA may not have a temperature in infection due to autonomic dysfunction.	Antibiotics may become less effective overtime. Patients may not wish to be admitted for intravenous antibiotics in later stages; discuss and document treatment preferences with patients and family.	Respiratory physiotherapy (suctioning and cough assist machines may help with secretion management if problematic) For urinary tract infections, low-dose prophylactic antibiotics or silver-lined catheters may reduce infection—refer to urology and continence services.
Blood pressure management	Non-pharmacological and pharmacological options	When there is symptomatic postural hypotension. Beware of supine hypertension.	Can pose significant challenges for example, syncope when opening bowels and postprandial BP drop, altered consciousness and risk of falls if still transferring or mobilising.	24-hour BP monitoring can inform management strategies. Specialist advice can be sought if needed.
Advance care planning and referral to specialist palliative care	Discussing treatments options, exploring personal preferences and recording these appropriately. Hospice services can provide practical and emotional support for person with MSA and their families and carers.	At all stages, by all healthcare professionals, may include Neurologist, Parkinsons’s nurse specialist, neurology nurse specialist, palliative care team and GP and primary care teams.	Advance care planning should be revisited regularly to ensure documentation is accurate, all relevant teams are aware and to ensure documentation of reconfirmation of wishes or changes to preferences recorded and communicated.	May include do not attempt resuscitation, Power of Attorney, advance directive for refusal of treatment, treatment preferences for interventions such as PEG or tracheostomy, where they would like to be cared for and end of life wishes, etc.

BP, blood pressure; CPAP, continuous positive airway pressure; GP, general practitioner; MSA, multiple system atrophy; NHS, National Health Service.

### Non-medical support for MSA patients and their families

People living with MSA and their families require support with practical, non-medical issues, especially as MSA is a rapidly progressive and untreatable disease, which significantly impacts on quality of life. This should be integrated into the conversations with patients and families who should be directed to relevant organisations for necessary support. The MSA Trust in the UK is an example (see box 2 for the potential role played by third sector organisations in MSA care). We recommend readers to find out about services locally available to their patients.

Box 2 **What are the main questions that people living with multiple system atrophy (MSA) ask and how the MSA Trust can help**

The MSA Trust supports anyone affected by MSA in either a personal or professional capacity. The charity works across the UK and the Republic of Ireland.

In 2019/2020, the charity’s main helpline, MSA nurse specialists and social welfare specialist collectively responded to over 24 000 calls and emails. The main reasons people contact the MSA Trust are:

- For good quality, reliable and up-to-date information on symptom management and living with MSA. The MSA Trust produces over 40 factsheets and information materials for people affected by MSA, including digital resources. It is accredited with the PIF Kitemark acknowledging a high standard of information produced. There is a series of webinars as well as resources for children and access to funded voice banking and a small welfare grant budget.
- To speak to a health and care professional who has knowledge of MSA and the difficulties associated with the condition. This will entail a referral to the MSA nurse specialists and/or social welfare specialist employed by the Trust.
- For peer support to feel less isolated when living with a rare disease. The MSA Trust runs physical and digital Support Groups, a HealthUnlocked online community, carers support group and an informal ‘drop in’ session regularly.
- For support and information for health and care professionals about MSA. The Trust provides tailored training sessions, an MSA Study Day and has specialist guides for the main professionals involved in the care of someone with MSA.
- For support with conversations that may not have been possible at diagnosis or in clinics such as planning for the future, emotional support and guidance, advice on obtaining care support, equipment advice, benefits information or general questions about life with MSA, for example, driving and holidays.

The MSA Trust can be contacted at support@msatrust.org.uk or on 0333 323 459

#### Welfare benefits and financial affairs

Patients and their carers are often negatively affected financially. They may have to stop working, fund their own care, equipment and housing adaptations and spend more on costs of daily living such as heating and transport. Many countries have a range of benefits (means and non-means tested) for people with MSA.

In certain countries (eg, England and Wales), people with complex healthcare needs may be entitled to non-means tested funding for supportive care within their own home or a nursing home. We recommend acknowledging early the impact that financial issues can have on the well-being of people with MSA, and directing them where possible to sources of support. This can include local third sector organisations (whether MSA specific or not) that can provide or signpost to advocacy services.

#### Arranging home adaptations and obtaining equipment

Early consideration of the requirement for home adaptations can help to maintain independence and assist carers. Patients need referral to occupational therapy services for assessment of the home environment and for provision of equipment and aids.

#### Planning ahead so their wishes are known and others can act on their behalf

It is important to consider this at an early stage when communication and mental capacity are not yet a concern. Patients and carers require information about Power of Attorney and other documentation that either allows others to be appointed to make decisions on a person’s behalf (in respect of managing property and financial affairs and/or making health and welfare decisions), or to document their wishes (eg, their will or with respect to their care).

### Case vignette: part 1 (fictional, based on clinical practice experience)

A 51-year-old woman, working in the broadcasting industry and previously well, was assessed by gastroenterology for abdominal pain and refractory constipation. A rigid colonoscopy was normal, and she was discharged with a diagnosis of irritable bowel syndrome. Two years later, she presented to urology with urinary incontinence, which she attributed to excess alcohol. However, shortly after, she developed daytime urgency. A bladder scan and urodynamics confirmed incomplete bladder voiding. Constant urgency persisted despite intermittent self-catheterisation five times daily and several medications for her bladder symptoms. Flexible cystoscopy showed bladder wall inflammation, and she received six different antibiotics sequentially and intravesical botulinum toxin was suggested. At the same time, her initial complaint of constipation was refractory to laxatives. Withing a few months, she consulted a cardiologist after fainting in hot weather. The cardiologist diagnosed orthostatic hypotension and discharged her with non-pharmacological management advice. She started developing social anxiety related to fainting, bowel and bladder symptoms. Her GP started her on antidepressants but without real benefit. She continued to faint, suffer from constipation and was performing intermittent self-catheterisation up to six times a day.

Eventually, she was assessed by neurourology who identified detrusor overactivity and incomplete bladder emptying. The neurourologist performed the first neurological examination, which was mostly normal. However, there was a subtle, occasional action right upper limb tremor with reduced right arm swing and brisk deep tendon reflexes. An anal sphincter EMG identified neurogenic changes in motor unit potentials. She was referred for specialist opinion for a possible mutisystem neurodegenerative condition.

### Case vignette: part 2

In the neurology clinic, she also described sleep problems and was sleeping in a different room from her husband as she would hit him while asleep, and shout and scream during vivid dreams. She had symptoms of Raynaud syndrome. She had stopped taking written notes due to illegibility and she sometimes bumped into things in the office. Her memory and thinking were normal, and she continued her job after workspace adaptations. Extensive autonomic function tests identified cardiovascular autonomic failure with orthostatic hypotension on head-up tilt and standing. She had an absent HR response to deep breathing, with abnormal blood pressure and HR responses during the Valsalva manoeuvre. Supine plasma catecholamine concentration was normal with no rise on being tilted. Her blood pressure profile was normal with occasional blood pressure falls on 24 hours monitoring. There was a clear exacerbation in blood pressure on poststand exercise. Neurological examination identified a subtle cerebellar syndrome with mild cerebellar atrophy on MR scan of brain. A diagnosis of possible MSA-C was established.

Shortly afterwards, she started collapsing during the day due to orthostatic hypotension. She started fludrocortisone; midodrine was later added as the symptoms persisted. The neurological examination showed only mild cerebellar and parkinsonian syndrome with brisk reflexes; however, the autonomic failure was severe and difficult to manage leading to recurrent falls and injuries. MR scan of brain showed moderate volume loss involving the pons and base of both middle cerebellar peduncles. A DAT scan showed reduced availability of presynaptic dopaminergic transporter, compatible with nigrostriatal degeneration. Her diagnosis was changed to probable MSA-C. After a frank and open conversation regarding the life expectancy, progression and prognosis, she put in place an advance directive of care and started voice banking; clearly expressed against having a tracheostomy or gastrostomy insertion in the later disease stages. She also signed consent for brain donation to the brain bank.

### Case vignette: part 3

Her condition deteriorated over the next 3 years with a combination of cerebellar ataxia, parkinsonian syndrome, pyramidal syndrome and severe autonomic dysfunction. A trial of levodopa gave no benefit and the orthostatic hypotension worsened and so the levodopa was stopped. During her illness, in addition to the movement disorder specialist, she was under the care of autonomic unit, neuro-urology, gastroenterology, specialist nurse, speech and language therapist, physiotherapist and occupational therapist. Within 6 years from onset, she was wheelchair-bound, needed hoisting for transfers, had unintelligible speech and dysphagia and had a suprapubic indwelling catheter. As she became increasingly dependent, she moved to live in a care home and received end-of-life care.

An autopsy was performed as she had consented to brain donation. Brain pathology showed a combination of striatonigral degeneration and olivopontocerebellar atrophy in the presence of glial cytoplasmic inclusion, confirming the definite diagnosis of MSA. A meeting was arranged with the family members and the neuropathology results communicated to them.

**Key points**

- Features suggesting possible prodromal multiple system atrophy (MSA) include unexplained genitourinary dysfunction, orthostatic hypotension, REM-sleep behaviour disorder and subtle parkinsonism/ataxia.
- Patients with suspected MSA should have a postvoiding residual urine scan, a lying and standing blood pressure, and an MR scan of brain with additional susceptibility-weighted and diffusivity images.
- A multidisciplinary team approach is essential to MSA patient care, with early involvement of clinical nurse specialists, physiotherapists, occupational health, speech and language, social welfare, and the continence team.
- Patients with MSA need regular medication rationalisation as they may not need several medications (eg, levodopa, blood pressure supportive medications) as the disease advances.
- Early discussion around MSA disease progression and advance care planning involving palliative care is important for all patients.

**Further reading**

- Wenning GK, Stankovic I, Vignatelli L, Fanciulli A, Calandra-Buonaura G, Seppi K, *et al*. The Movement Disorder Society Criteria for the Diagnosis of Multiple System Atrophy. Vol. 37, Movement Disorders. John Wiley and Sons Inc; 2022. p. 1131–48.
- Poewe W, Stankovic I, Halliday G, Meissner WG, Wenning GK, Pellecchia MT, *et al*. Multiple system atrophy. Vol. 8, Nature reviews. Disease primers. NLM (Medline); 2022. p. 56.
- The FAQ sections on the MSA Trust and MSA Coalition websites for an understanding of patient concerns.

## Data Availability

Data sharing not applicable as no datasets generated and/or analysed for this study.
